# Chronic Effects of Palmitate Overload on Nutrient-Induced Insulin Secretion and Autocrine Signalling in Pancreatic MIN6 Beta Cells

**DOI:** 10.1371/journal.pone.0025975

**Published:** 2011-10-05

**Authors:** Maria L. Watson, Katherine Macrae, Anna E. Marley, Harinder S. Hundal

**Affiliations:** 1 Division of Cell Signalling and Immunology, Sir James Black Centre, College of Life Sciences, University of Dundee, Dundee, United Kingdom; 2 AstraZeneca, CVGI, Alderley Park, Macclesfield, Cheshire, United Kingdom; Florida International University, United States of America

## Abstract

**Background:**

Sustained exposure of pancreatic β cells to an increase in saturated fatty acids induces pleiotropic effects on β-cell function, including a reduction in stimulus-induced insulin secretion. The objective of this study was to investigate the effects of chronic over supply of palmitate upon glucose- and amino acid-stimulated insulin secretion (GSIS and AASIS, respectively) and autocrine-dependent insulin signalling with particular focus on the importance of ceramide, ERK and CaMKII signalling.

**Principal Findings:**

GSIS and AASIS were both stimulated by >7-fold resulting in autocrine-dependent activation of protein kinase B (PKB, also known as Akt). Insulin release was dependent upon nutrient-induced activation of calcium/calmodulin-dependent protein kinase II (CaMKII) and extracellular signal-regulated kinase (ERK) as their pharmacological inhibition suppressed GSIS/AASIS significantly. Chronic (48 h, 0.4 mM) palmitate treatment blunted glucose/AA-induced activation of CaMKII and ERK and caused a concomitant reduction (∼75%) in GSIS/AASIS and autocrine-dependent activation of PKB. This inhibition could not be attributed to enhanced mitochondrial fatty acid uptake/oxidation or ceramide synthesis, which were unaffected by palmitate. In contrast, diacylglycerol synthesis was elevated suggesting increased palmitate esterification rather than oxidation may contribute to impaired stimulus-secretion coupling. Consistent with this, 2-bromopalmitate, a non-oxidisable palmitate analogue, inhibited GSIS as effectively as palmitate.

**Conclusions:**

Our results exclude changes in ceramide content or mitochondrial fatty acid handling as factors initiating palmitate-induced defects in insulin release from MIN6 β cells, but suggest that reduced CaMKII and ERK activation associated with palmitate overload may contribute to impaired stimulus-induced insulin secretion.

## Introduction

Hyperlipidemia is one of a cluster of abnormalities associated with the metabolic syndrome, which not only promotes insulin resistance, but results in the dysfunction of numerous cellular responses in tissues such as skeletal muscle [1], heart [2], liver [3], adipose [4] and the pancreas [5]. In the pancreas, for example, it has been demonstrated that during the pre-diabetic and diabetic states, there is an increase in intracellular fatty acids [6] that desensitise pancreatic beta cells to glucose [7]. An important consequence of this reduced glucose “sensing” capacity is a reduction in glucose-stimulated insulin secretion (GSIS), which contributes to the impaired glucose homeostasis associated with the diabetic state.

Fatty acids are thought to play an essential role in GSIS augmenting the glucose-induced secretion of insulin [8], [9]. During the fasting state, fatty acids are free in the cytosol of beta cells, and, under these circumstances, are channelled into mitochondria for β-oxidation and generation of ATP [10] and do not promote any detectable increase in insulin secretion. However, upon feeding, the rise in blood glucose not only promotes insulin release from beta cells by a mechanism involving the inactivation (closure) of plasma membrane K^+^_ATP_ channels, but glucose will also contribute to metabolic anaplerosis. Citrate produced in the mitochondria from glucose metabolism will form malonyl-CoA in the cytosol [11], [12], which prevents β-oxidation by inhibiting carnitine palmitoyltransferase (CPT-1), thereby allowing an increase in long chain fatty acids which can stimulate insulin secretion. Although the precise mechanism is unknown it is thought that long chain fatty acids can either modulate intracellular targets that stimulate insulin release [13], [14] or form complex lipids, such as diacylglycerol (DAG), that interact with insulin granule proteins leading to granule fusion with the membrane [15]. In addition, the presence of free fatty acids, supplied by diet or circulating unbound free fatty acids in the aqueous phase, has been suggested to activate the fatty acid G-protein coupled receptor (GPR40). Activation of GPR40 causes an increase in intracellular Ca^2+^, which is thought to be induced *via* activation of the Gαq-phospholipase C pathway. The increase in free cytosolic Ca^2+^ plays a crucial role in stimulating insulin secretion [14].

Although important for beta cell function, sustained increases in fatty acid availability and influx, as occur in response to high fat feeding [6], can induce both dysfunction [7] and death of beta cells [16] and thereby contribute to the pathogenesis of diabetes mellitus. Sustained exposure of pancreatic beta cells to fatty acids, such as palmitate, has been linked to a loss in GSIS and increased apoptosis [7], [17]–[20]. What is less well understood is the mechanism underpinning the lipotoxic effects of palmitate. Analysis of mice lacking GPR40 indicate that although important in mounting acute responses to fatty acid supply the receptor is unlikely to contribute to pancreatic dysfunction induced in response to sustained increases in fatty acid availability [14]. While there is no compelling evidence implicating other fatty acid receptors or transporters an increase in fatty acid provision has been suggested to disrupt the glucose-fatty acid cycle by inhibition of pyruvate dehydrogenase, which switches fuel consumption from glucose to fatty acid oxidation [18], [21]. Fatty acids may also induce much greater oxidation by increasing the expression of CPT-1 [22] whilst decreasing that of acetyl CoA carboxylase [23]. This exaggerated fatty acid oxidation may promote beta cell insensitivity to glucose resulting in a concomitant reduction in GSIS, which may also be underpinned by defects in the regulation or expression of transcription factors, such as PDX-1 [24], or indeed that of the insulin gene itself [25]. In many cell types increased provision of fatty acids has been linked to enhanced intracellular synthesis and accumulation of ceramide, a sphingolipid that can be generated *de novo* from saturated fatty acids (e.g. palmitate) *via* the seine palmitoyl transferase (SPT) pathway. Ceramide has been implicated strongly in cell apoptosis and in the pathogenesis of insulin resistance and there is evidence in the literature suggesting that it may be an effector molecule mediating palmitate-induced beta cell dysfunction [26], [27] and beta cell death [6].

Here we show that sustained exposure of pancreatic MIN6 beta cells to palmitate results in a substantial loss in insulin secretion in response to glucose or amino acid provision. This fatty acid-induced impairment in insulin secretion blunts autocrine signalling by the hormone, but, unlike in other cell types, has no detectable effect on PKB-directed insulin signalling in response to exogenously supplied hormone. Furthermore, we demonstrate that the palmitate-induced reduction in insulin secretion cannot be attributed to ceramide action, but may be due, in part, to a loss in nutrient-induced CaMKII and ERK signalling which normally supports the stimulus-dependent secretion of insulin.

## Results

### Palmitate induces a loss in glucose- and amino acid-stimulated insulin secretion and induces apoptosis in MIN6 β cells

**Figure 1:**
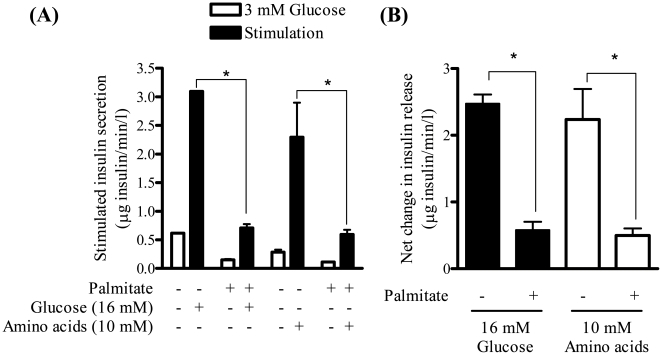
Palmitate induces a loss in glucose- and amino acid-stimulated insulin secretion. (A) MIN6 β cells were incubated with palmitate (0.4 mM) for 48 h prior to incubation with glucose (3 mM or 16 mM) or with an amino acid/glucose mix (10 mM leucine and glutamine/3 mM glucose) for 1 h. Glucose-stimulated insulin secretion (GSIS) and amino acid-stimulated insulin secretion (AASIS) were then assessed as described in the methods. The net change in insulin secretion from 3 to 16 mM glucose or to 10 mM amino acid was analysed and graphed (B). Values are mean ± SEM from 3 separate experiments. The asterisk signifies a significant difference from the appropriate control (P<0.05).

Insulin release from pancreatic β cells is normally negligible in the presence of fasting blood glucose levels (i.e. ∼3–4 mM). However, in response to an increase in extracellular glucose (as occurs during the post absorptive phase when it may rise by 2–4 fold, i.e. 7–16 mM) or indeed in response to increased amino acid provision, secretion of insulin is enhanced significantly [28]. This glucose- and amino acid-dependent response is illustrated in Figure 1A, which shows a significant increase (up to 7.5-fold) in insulin secretion when MIN6 β cells were incubated in media containing 16 mM glucose or a 3 mM glucose/10 mM leucine and glutamine mix (shown previously to stimulate insulin secretion independently of glucose metabolism [29]). Irrespective of the stimulus used, this nutrient-dependent increase in insulin secretion was significantly blunted when MIN6 β cells were preincubated with palmitate (0.4 mM) for a period of 48 h (Figure 1A) falling by ∼75% when expressed as a net change (Figure 1B). Intriguingly, even in the absence of a nutrient stimulus we observed a significant reduction in basal insulin secretion indicating that palmitate over supply may also target the secretory process that operates in response to a sub-threshold glucose (3 mM) stimulus.

**Figure 2:**
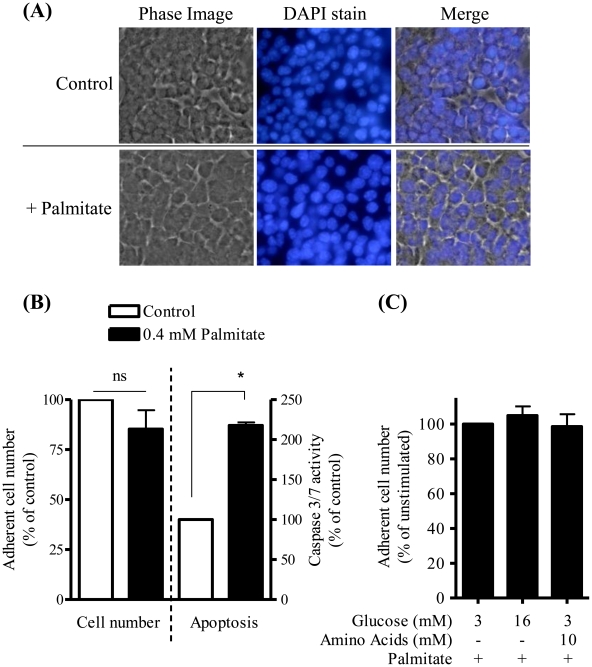
Effects of palmitate on MIN6 β cell number and apoptosis. MIN6 β cells were incubated in the absence or presence of palmitate (0.4 mM) for 48 h prior to analysis of cell number by DAPI staining (A) or (B) caspase 3/7 activity (a proapoptotic marker). Three random visual fields from duplicate samples were chosen (typical phase, DAPI stained and merged images are shown in panel A), and adherent cell number noted from control or palmitate-treated cells following DAPI staining (A and B). Alternatively, following the fatty acid incubation period cells were incubated with caspase 3/7 assay reagent and caspase 3/7 activity determined by luminescence as described in methods (B). In some experiments, cells that had been pre-treated with palmitate (0.4 mM, 48 h) were subsequently incubated with glucose (16 mM) or with an amino acid/glucose mix (10 mM leucine and glutamine/3 mM glucose) for 1 h prior to analysis of cell number by DAPI staining (C). Bar values are mean ± SEM from 3 or 4 separate experiments. The asterisk signifies a significant difference from the appropriate control value (P<0.05).

Given that palmitate is known to induce apoptosis of β cells it is plausible that the loss in GSIS may be related to a reduction in MIN6 cell number. However, analysis of adherent cell numbers based on DAPI staining did not reveal any significant changes in control or palmitate-treated cell populations (Figure 2A and 2B) and, in separate experiments, we could not detect any additional effect upon cell viability of subjecting palmitate-treated MIN6 cells to a glucose or amino acid stimulus suggesting that cells were able to tolerate the combind stress of palmitate exposure and energy stimulation (Figure 2C). Nonetheless, it is noteworthy that palmitate treatment induces a 2-fold increase in caspase 3/7 activity (a proapoptotic marker) in MIN6 cells (Figure 2B), suggesting that whilst apoptosis had been initiated by the fatty acid this had not yet translated to a reduction in cell number over the 48 h incubation period being studied. However, it is noteworthy that in preliminary experiments analysis of cell number following 60 h of treatment with palmitate had revealed a significant decline in cell survival; an observation that we had used to help to inform our choice of using a shorter palmitate incubation period for the studies reported in this paper (data not shown).

### The loss in GSIS cannot be attributed to ceramide action

**Figure 3:**
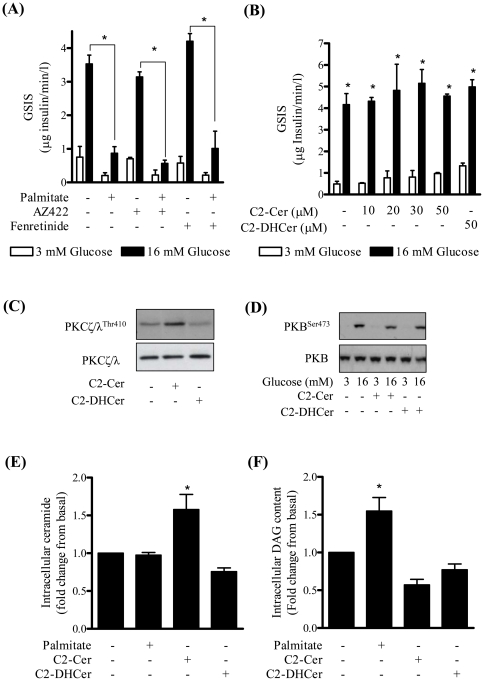
The *de novo* ceramide synthesis pathway is not involved in the palmitate-induced loss in glucose-stimulated insulin secretion. MIN6 β cells were incubated either with or without 10 µM AZ422 (a SPT inhibitor) or 5 µM fenretinide (an inhibitor of dihydroceramide desaturase) (A) for 48 h or with varying concentrations of ceramide and dihydroceramide (B–F) for 2 h. Palmitate (0.4 mM) was present for 48 h where indicated. Glucose was added for 1 h for analysis of GSIS (A and B). Alternatively, cells were either lysed for immunoblot analysis using antibodies against (C) PKCζ/λ^Thr410^ and total PKCζ/λ (used as loading control), (D) PKB^Ser473^ and total PKB (as a loading control) or harvested and lipids extracted to assess (E) ceramide or (F) DAG content. Bars represent mean ± SEM of 3 experiments and asterisks signify a significant difference from the untreated control value (P<0.05).

Palmitate-driven ceramide synthesis has been shown to impair insulin action and induce apoptosis in a number of different cell types and evidence exists in the literature implicating ceramide in β-cell dysfunction. To test whether ceramide may be an effector molecule mediating the lipid-induced loss in GSIS in MIN6 β cells we investigated the effect of two inhibitors that suppress *de novo* ceramide synthesis, AZ422 and fenretinide. AZ422 inhibits the activity of SPT (Figure S1) and fenretinide suppresses the conversion of dihydroceramide to ceramide by inhibiting dihydroceramide desaturase. Both inhibitors failed to antagonise the palmitate-induced loss in GSIS indicating that any increase in ceramide synthesis was unlikely to account for the impaired secretion of insulin in response to palmitate (Figure 3A). Consistent with this proposition, raising cell ceramide content by incubating MIN6 β cells with increasing concentrations of C2-ceramide (a short-chain cell permeant ceramide analogue) did not alter GSIS when compared to output of insulin from untreated cells or those incubated with the inert C2-dihydroceramide analogue (Figure 3B). Ceramide concentrations above 50µM were not used as these proved cytotoxic. It is important to stress that whilst 50µM C2-ceramide did not affect GSIS this concentration of ceramide was effective in promoting phosphorylation/activation of atypical PKCs (a known ceramide target), whereas the inactive C2-dihydroceramide did not (Figure 3C). In both adipocytes and muscle cells ceramide is a potent inhibitor of PKB/Akt activation [30], [31]. However, it is noteworthy that in MIN6 β cells activation of PKB in response to GSIS was unaffected by cell incubation with C2-ceramide (Figure 3D). Analysis of cell ceramide content following a 48 h palmitate incubation unexpectedly revealed that there was little, if any, detectable ceramide synthesis from palmitate in MIN6 β cells (Figure 3E). The DAG kinase assay that we employed readily detected significant increases in cell ceramide following cell treatment with C2-ceramide (Figure 3E) and in palmitate-driven DAG synthesis (Figure 3F). Incubation with C2-dihyrdoceramide did not register any increase in ceramide consistent with the fact that it does not serve as a substrate for DAG kinase (Figure 3E).

### Effects of palmitate on autocrine insulin signalling in MIN6 β cells

**Figure 4:**
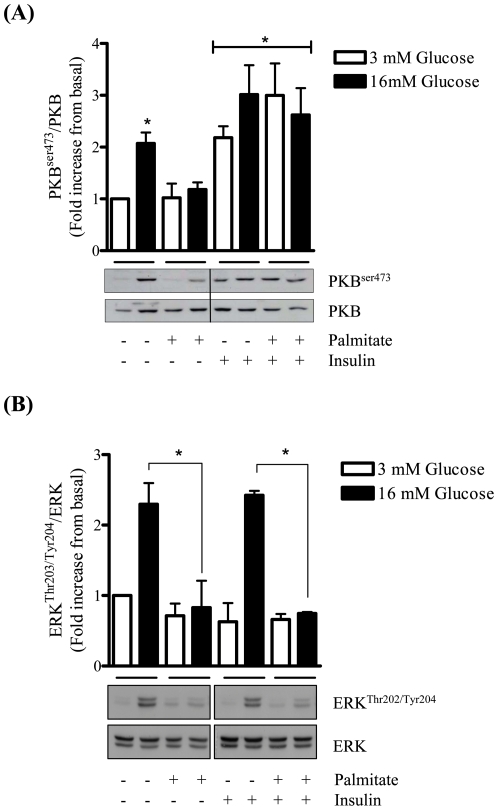
Palmitate does not inhibit PKB activation in response to exogenously added insulin but does prevent glucose-induced activation of ERK. MIN6 β cells were incubated in the absence or presence of palmitate (0.4 mM) for 48 h. Glucose (16 mM) was added for 1 h and insulin (100 nM) in the penultimate 10 min of this incubation period. Cells were harvested and immunoblotted for phospho-PKB^Ser473^ (A) and phospho-ERK^Thr202/Tyr204^ (B) and normalised against total protein. Bars represent mean ± SEM from 3 separate experiments and asterisks signify a significant difference from the untreated control values (P<0.05).

The effect of palmitate on insulin signalling was subsequently investigated in MIN6 β cells. Under conditions of low (3 mM) glucose the phosphorylation of PKB and that of ERK1 and ERK2 was not readily detectable, but was enhanced significantly upon incubation of MIN6 β cells with high (16 mM) glucose (Figure 4A and 4B). This glucose-induced increase in PKB and ERK phosphorylation was blunted significantly upon preincubation of MIN6 β cells with palmitate (Figure 4A and 4B). To assess whether this palmitate-induced loss in PKB and ERK phosphorylation was due to impaired GSIS, insulin was added exogenously to the media bathing MIN6 β cells in the absence and presence of palmitate. This experimental approach revealed that palmitate was unable to blunt the activation of PKB in response to exogenously added insulin (Figure 4A) and that the impaired activation of PKB seen in response to high glucose was more likely explained by the inhibitory effect of palmitate on insulin secretion rather than insulin signalling *per se*. In striking contrast, exogenously supplied insulin did not activate ERK and was unable to over-ride the inhibitory effect of palmitate on glucose-stimulated ERK phosphorylation (Figure 4B). This latter observation clearly implies that glucose-induced ERK activation is not reliant upon autocrine activation of proximal insulin-signalling components and depends upon a mechanism that is distinct from that promoting activation of PKB.

### Activation of ERK and CaMKII is required to support glucose- and amino acid-induced insulin secretion

**Figure 5:**
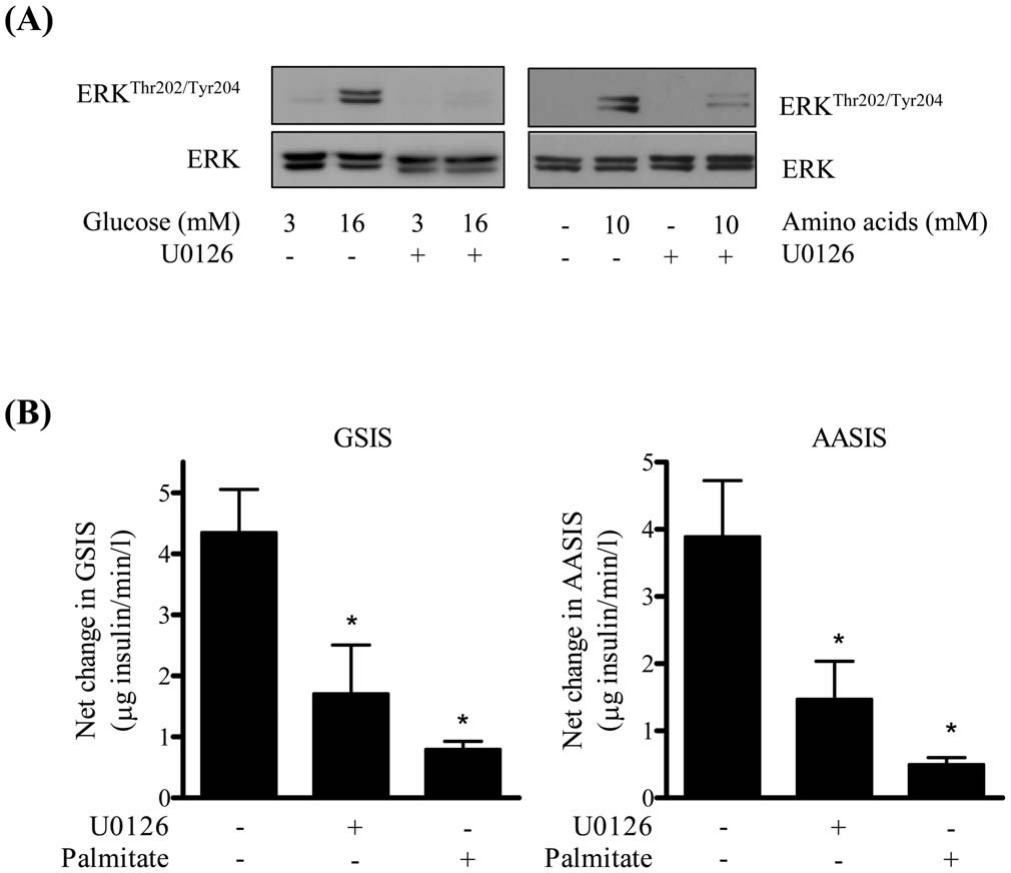
ERK inhibition induces a loss in glucose-stimulated insulin secretion (GSIS) and amino acid-stimulated insulin secretion (AASIS). MIN6 β cells were incubated in the absence or presence of 20 µM U0126, as indicated prior to being exposed to either 3 mM or 16 mM glucose for 1 h or alternatively to an amino acid stimulus (10 mM leucine/glutamine and 3 mM glucose). At the end of this incubation cells were (A) lysed for analysis of ERK phosphorylation in whole cell lysates or (B) prepared for analysis of net changes in GSIS or AASIS (B) as described in methods. Bars represent mean ± SEM from 3 separate experiments and asterisks signify a significant difference from the untreated control values (P<0.05).

The finding that palmitate blunts glucose-stimulated ERK signalling and also induces an attendant reduction in insulin secretion implies that activation of ERK may be required to support stimulus-induced insulin secretion. To test this proposition we assessed the effect of U0126, a selective inhibitor of the MEK-ERK signalling cascade, upon both glucose- and amino acid-induced ERK activation and insulin secretion. Figure 5A shows that increased provision of glucose or amino acids induces activation of ERK signalling, which was suppressed by coincubation of cells with U0126. Associated with this inhibition in ERK activation by U0126 was a significant reduction (by up to 62%) in insulin secretion from MIN6 β cells in response to increased availability of both glucose and amino acids (Figure 5B).

**Figure 6:**
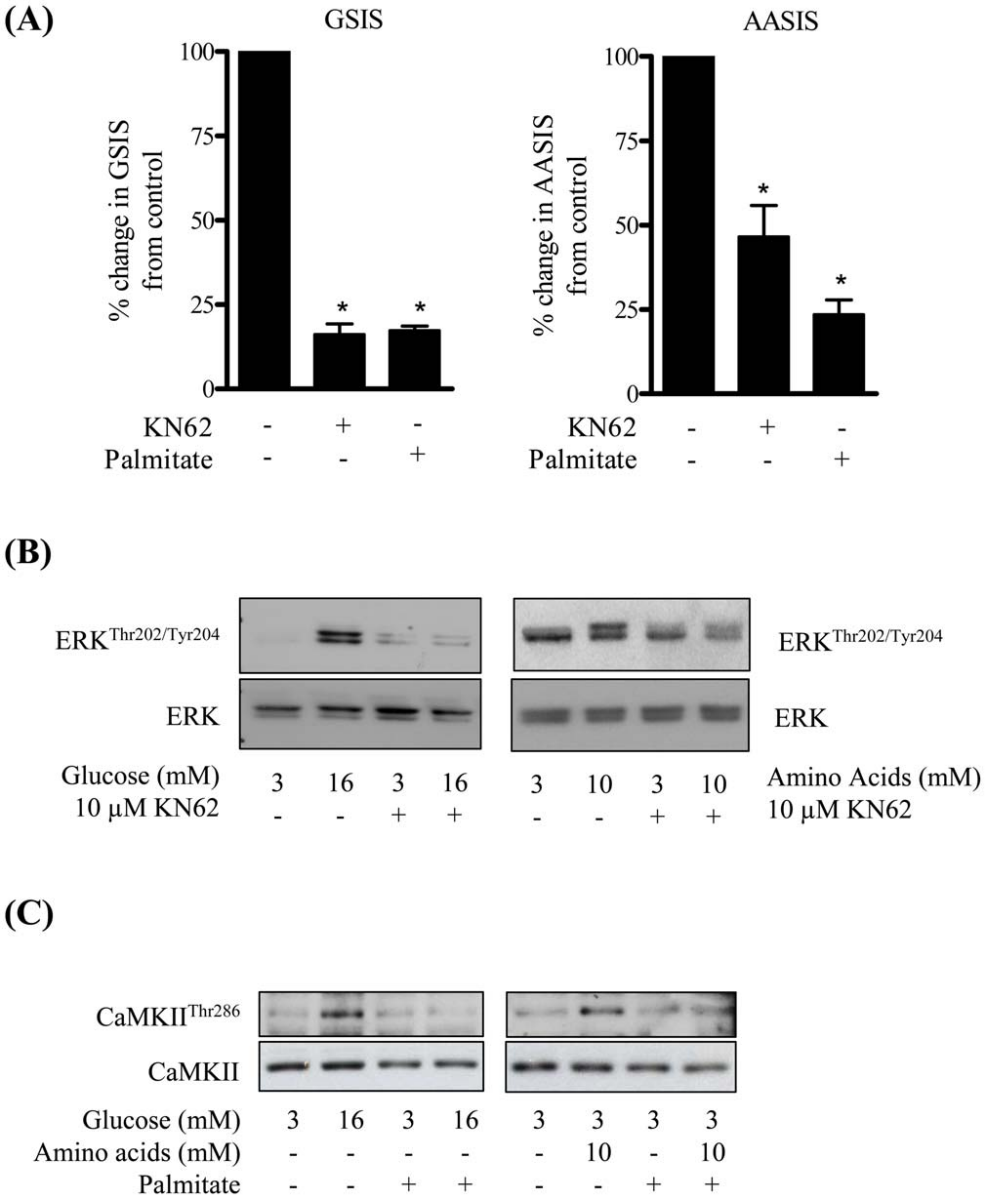
Palmitate antagonises the stimulus-induced activation of ERK and CaMKII in MIN6 β cells. MIN6 β cells were incubated with 10 µM KN62 (an inhibitor of CaMKII) for 2 h. In the last hour of this incubation cells were incubated with either glucose (16 mM) or amino acids (10 mM) prior to assessing (A) GSIS or AASIS, (B) ERK activation by analysis of ERK^Thr202/Tyr204^ phosphorylation. Total ERK content was used for assaying protein loading. Alternatively, MIN6 β cells were incubated in the absence or presence of palmitate (0.4 mM) for 48 h and then stimulated insulin secretion assessed by the addition of glucose or amino acids for 1 h at the concentrations indicated. Cells were lysed and lysates immunoblotted to assay phospho-CaMKII^Thr286^ phosphorylation with native CaMKII serving as a gel loading control (C). Bars represent mean ± SEM from 3 separate experiments and asterisks signify a significant difference from the untreated control values (P<0.05).

Previous work has indicated that glucose-induced increases in cytosolic calcium can activate the ERK pathway *via* stimulation of CaMKII and that this activation occurs independently of Ras and Raf [32]. We therefore assessed the effects of CaMKII inhibition on both glucose-induced ERK activation and insulin secretion. Figure 6A and 6B shows that KN-62, a CaMKII inhibitor, antagonised both ERK activation and insulin secretion in response to increased glucose and amino acid provision. The notion that CaMKII may serve as an intermediary in the activation of ERK signalling and GSIS is further strengthened by the observation that phosphorylation/activation of CaMKII itself in response to glucose and amino acids was reduced following palmitate treatment (Figure 6C).

### Impaired GSIS and ERK1/2 signalling in MIN6 β cells is not dependent upon fatty acid oxidation

**Figure 7:**
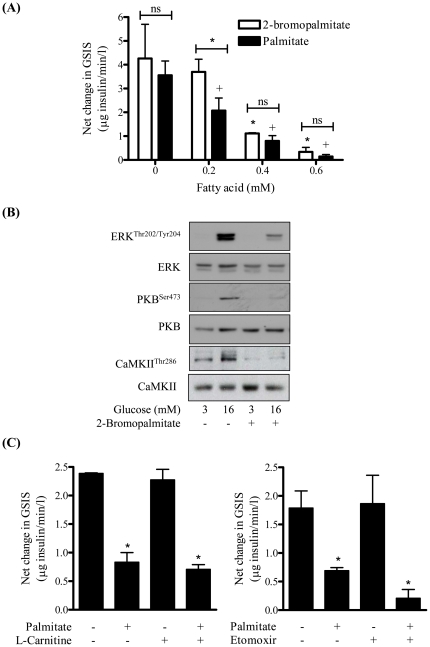
The repressive effects of palmitate on glucose-stimulated insulin secretion, PKB and ERK activation are not dependent upon fatty acid oxidation. (A) MIN6 β cells were incubated with varying concentrations of palmitate and 2-bromopalmitate (48 h) as indicated. Glucose (16 mM) was added for 1 h during serum starvation and the net change in glucose-stimulated insulin secretion assessed. (B) Cells were incubated in the absence or presence of 0.4 mM 2-bromopalmitate for 48 h and with glucose at the indicated concentration for 1 h. Cells were lysed and lysates immunoblotted for analysis of ERK^Thr202/Tyr204^, PKB^Ser473^, CaMKII^Thr286^ and total PKB, ERK and CaMKII. (C) Cells were incubated in the absence or presence of palmitate (0.4 mM) for 48 h and then after serum starvation cells were exposed to either L-carnitine (1 mM) or etomoxir (0.2 mM) for 2 h as indicated. Glucose (16 mM) was added during the final hour to induce GSIS and media removed for analysis of insulin content. Bars represent mean ± SEM from 3 separate experiments and asterisks or a cross signify a significant difference from the untreated control values or between indicated bars (P<0.05).

To assess whether the suppressive effect of palmitate upon ERK signalling and GSIS involves mitochondrial fatty acid metabolism we investigated the effect of 2-bromopalmitate, a non-oxidisable analogue of palmitate. Figure 7A shows that 2-bromopalmitate proved just as effective as palmitate in suppressing GSIS and, despite its lack of metabolism, was just as effective in suppressing activation of ERK, PKB and CaMKII (Figure 7B). To further substantiate that fatty acid oxidation was unlikely to be an important determinant of the palmitate-induced loss in GSIS, MIN6 β cells were incubated with either carnitine or etomoxir. Carnitine provision has been shown to enhance fatty acid oxidation, whereas etomoxir targets carnitine palmitoyltransferase I (CPT1) and blocks mitochondrial fatty acid oxidation. Figure 7C shows that carnitine supplementation had no impact on palmitate's inhibitory effect on GSIS, whereas blocking mitochondrial fatty acid uptake using etomoxir caused a marginal enhancement in palmitate's repressive effect on GSIS.

## Discussion

Sustained increases in circulating free fatty acids is an early indicator of insulin resistance [33]. As with skeletal muscle and other insulin sensitive tissues, pancreatic beta cells are susceptible to the negative effects of fatty acid excess [34]. Whilst fatty acids are essential for supporting GSIS, increased availability as seen in obesity can overload β cells, prompting cellular dysfunction as characterised by a reduction in GSIS [7] and increased susceptibility to lipoapoptosis [35]. An increase in long chain CoAs and palmitate-derived lipids such as ceramide and DAG has been demonstrated in β cells of diabetic rodent models [6]. Ceramide has been widely implicated as a pro-apoptotic agent and given that it can be generated from palmitate, a plausible hypothesis is that pancreatic β-cell dysfunction and apoptosis is mediated by palmitate-driven ceramide synthesis. It thus follows that suppressing cellular production of ceramide may potentially have beneficial consequences with regard β cell function. However, our analysis has revealed that whilst sustained provision of palmitate impairs GSIS from MIN6 β cells the fatty acid does not appear, under these circumstances, to drive an increase in ceramide synthesis. MIN6 β cells express both subunits of serine palmitoyl transferase (SPT) and whilst we find the enzyme is competent in generating ceramide from palmitate *in vitro* (see Figure S1) our observations indicate that ceramide is unlikely to be a key effector underpinning the loss in GSIS in response to palmitate in intact MIN6 β cells. The finding that inhibiting *de novo* ceramide synthesis *via* SPT or indeed incubating cells with a cell permeant ceramide analogue had no effect on GSIS would be consistent with this proposition. Moreover, it is noteworthy that our observations are fully in line with very recent studies in MIN6 cells in which sustained exposure to palmitate failed to induce increases in intracellular ceramide synthesis [36] or in which inhibition of *de novo* ceramide synthesis [37] did not attenuate the inhibitory effect of palmitate on insulin secretion.

An important finding to emerge from our work is that, unlike adipocytes and skeletal muscle cells [30], [31], palmitate does not impair insulin signalling in MIN6 β cells given that prior incubation of cells with palmitate had no detectable effect on activation of PKB signalling in response to exogenously added insulin. In both fat and muscle cells the loss in insulin-signalling capacity associated with palmitate supply is mechanistically attributed to ceramide generated from the fatty acid [30], [31]. However, such a mechanism appears not to operate in MIN6 β cells based on the finding that C2-ceramide does not inhibit PKB activation in response to a raised glucose stimulus, which invokes GSIS and autocrine signalling by insulin in these cells. Although C2-ceramide appears not to modulate GSIS in our hands, recent work has suggested that the lipid does inhibit glucose-induced insulin gene expression in MIN6 β cells [38]. It is therefore possible that the effects of ceramide may be selectively targeted at insulin gene expression rather than hormone secretion, but given that we detect very little *de novo* synthesis of ceramide from palmitate the importance of such regulation may only become germane if the lipid is generated in significant amounts *via* other routes involving, for example, upregulation in the activity of membrane bound sphingomyelinases [39].

Whilst insulin is a very poor stimulus for inducing ERK activation in MIN6 β cells, cell incubation with high glucose (16 mM) leads to a robust activation/phosphorylation of ERK. Our findings indicate that activation of ERK signalling is important for supporting efficient GSIS/AASIS based on sensitivity to the MEK inhibitor, U0126. This inhibitor has been reported to promote activation of AMPK in HEK293 cells and so it is plausible the drug-induced reduction in GSIS/AASIS may be due to AMPK-mediated antagonism [40]. However, we believe this is highly unlikely given that U0126 has no direct effect on AMPK activity in cell-free assays [41] and does not promote any detectable increase in phosphorylation of acetyl CoA carboxylase, a physiological downstream AMPK substrate in MIN6 cells (data not shown).

Previous work has shown that the sequalae of events that follow uptake and metabolism of glucose (i.e. increased cytoplasmic ATP/ADP ratio, closure of ATP-sensitive K^+^ channels, depolarization of the cell membrane, opening of voltage-gated calcium channels and the subsequent influx of extracellular calcium into the cell) induces activation of CaMKII, which acts upstream of MEK1/2 to mediate ERK phosphorylation [32], [42]. ERK activation in turn has been reported to promote cytoskeletal rearrangement [43] and phosphorylation of cytoplasmic proteins implicated in exocytosis of insulin granules, such as synapsin I [44]. However, under conditions when β cells are chronically exposed to an elevated supply of palmitate the glucose-induced activation of ERK is significantly blunted. We believe that the reduced activation of ERK is likely to be a consequence of an associated reduction in CaMKII signalling. This belief is based not only on the finding that palmitate impairs the glucose-induced activation of CaMKII, but the observation that, even in cells not exposed to the fatty acid, CaMKII inhibition with KN-62 suppresses activation of ERK in response to a raised glucose stimulus. Moreover, consistent with the idea that CaMKII-directed signaling is important for promoting insulin secretion we find that KN-62 causes a significant reduction in GSIS. In addition to glucose, certain amino acids are also potent insulin secretagogues being able to induce hormone secretion as a consequence of their capacity to depolarise the β-cell membrane and cause the concomitant opening of voltage-gated calcium channels. Membrane depolarisation may be triggered directly by some amino acids (e.g. arginine), whereas others (e.g. alanine, glutamine, leucine) may induce it *via* a combination of their influx into β-cells *via* Na-coupled amino acid transporters and/or metabolism that enhances ATP generation with subsequent effects upon K-ATP channels in much the same manner as glucose [45]. Given that the effects of glucose and amino acids converge upon regulation of a common plasma membrane channel it is perhaps not unsurprising that sustained exposure of MIN6 β cells to palmitate also results in impaired downstream activation of CaMKII and ERK in response to amino acid provision.

A key issue of interest is the mechanism by which over supply of palmitate induces impaired GSIS. Since metabolism of glucose and the associated increase in cytoplasmic ATP/ADP ratio is central for stimulus-secretion coupling in β-cells it is plausible that palmitate may reduce glucose metabolism and thereby limit GSIS. However, previous work has shown that sustained (72 h) incubation of MIN6 β cells with palmitate does not affect glycolytic flux or ATP synthesis [46] negating this as a likely possibility. It also appears unlikely that oxidation of palmitate itself is required for initiating the reduction in GSIS. Etomoxir, which inhibits mitochondrial fatty acid uptake *via* CPT1, failed to prevent the reduction in GSIS, whereas carnitine supplementation, which would promote mitochondrial fatty acid uptake/oxidation [47], did not augment the palmitate-induced loss in insulin secretion. Furthermore, the finding that 2-bromopalmitate, which cannot undergo mitochondrial oxidation, was as potent as palmitate in suppressing GSIS underscores the notion that changes in mitochondrial fatty acid oxidation are unlikely to account for the observed reduction in GSIS. This latter observation is also in line with work assessing the inhibitory effect of palmitate on glucose-induced insulin gene expression in β cells in which 2-bromopalmitate was found to mimic the repressive effect of palmitate and in which fatty acid esterification rather than oxidation was deemed as the more important determinant of palmitate-induced lipotoxicity [48]. Consistent with this suggestion we did observe that the repressive effect of palmitate upon GSIS was further enhanced in the presence of etomoxir, which may promote greater partitioning of palmitate into pathways favouring its esterification to diacylglycerol (DAG) and triglyceride. While we did not detect significant generation of ceramide from palmitate in MIN6 β cells synthesis of DAG was indeed elevated in palmitate-treated MIN6 β cells. An increase in intracellular DAG would be expected to promote activation of DAG-sensitive PKCs, which are thought to mediate the effects of acute increases in fatty acid provision upon numerous aspects of β-cell function, including potentiation of GSIS [49]. However, it is plausible that sustained fatty acid overload may result in synthesis and accumulation of distinct DAG species and/or activation of specific PKC isoforms that are potentially pathogenic in nature. Cantley and coworkers have suggested that PKCε may contribute to impaired GSIS given that its deletion in mouse β-cells induces amplification of pathways stimulating GSIS in isolated islets cultured chronically in the presence of palmitate [50]. Whilst the increase in GSIS induced in PKCε knock-out islets was suggested to operate independently of any changes in intracellular calcium, very recent work has reported that sustained exposure of islets to palmitate impairs calcium influx at membrane domains that are in close proximity to insulin granules with a consequential reduction in their exocytosis [51]. Using a fluorescent calcium indicator dye, Oregon-Green-BAPTA-1, we find that glucose induces calcium transients in MIN6 β cells and that these are muted in cells that have been chronically pre-exposed to palmitate for 48 hours (see Figure S2). It is plausible that these calcium transients may normally support the activation of CaMKII and that their reduction impairs efficient activation of this kinase with a “knock-on” effect upon downstream targets involved in the insulin secretory process. Testing this latter possibility represents an important investigative goal of future work.

In summary, whilst the lipotoxic effects of fatty acid over load will undoubtedly promote metabolic (endoplasmic reticulum and oxidative) stress and reduce the insulin secretion capacity of beta cells *via* activation of pro-apoptotic pathways [45], our studies indicate that insulin secretion from MIN6 β cells in response to glucose or amino acids is critically dependent upon the nutrient-induced activation of CaMKII and ERK. Sustained exposure of MIN6 β cells to palmitate results in impaired activation of both signalling molecules with an attendant reduction in insulin secretion. While there is no suggestion from our work that the repressive effects of palmitate are mediated by an increase in fatty acid-derived ceramide synthesis or fatty acid oxidation, the loss in stimulus-secretion coupling may involve a targeted disruption in calcium influx *via* voltage-dependent calcium channels in response to increased esterification/synthesis of fatty acid-derived metabolites such as DAG.

## Methods

### Reagents and antibodies

Dulbeccos Modified Eagle Medium (DMEM), foetal bovine serum (FBS), Trypsin/EDTA solution and penicillin/streptomycin solution (containing penicillin G sodium (10,000 unit/ml), streptomycin sulphate (10,000 µg/ml) in 0.85% saline) were purchased from Gibco/Invitrogen Life Technologies (Paisley, UK). Other reagent grade chemicals were purchased from Sigma-Aldrich (Poole, UK). This included insulin, palmitate, C2-dihydroceramide, fenretinide, 2-bromopalmitate, dimethyl sulphoxide (DMSO), 4,6-diamino-2-phenylindole diacetate (DAPI) and anti-actin. Bovine Serum Albumin (BSA) for conjugating palmitate and complete protein phosphatases inhibitor tablets were purchased from Boehringer-Roche Diagnostics (Basel, Switzerland). The Caspase-Glo 3/7 assay kit for apoptosis analysis was purchased from Promega (Southampton, UK). The ELISA (Enzyme-Linked ImmunoSorbent Assay) kit for insulin detection in MIN6 β cells was from Mercodia (Uppsala, Sweden). C2-ceramide was purchased from Tocris (Bristol, UK). Antibodies to Protein Kinase B (PKB)/Akt, PKB/Akt phospho-Ser^473^ ERK phospho-Thr^202^/Tyr^204^, CaMKII phospho-Thr^286^ and CaMKII were purchased from New England Biolabs (Herts, UK) whilst that to atypical PKC λ/ζ was from Santa Cruz (California, USA). The inhibitor AZ422 was synthesised and kindly supplied by Astra Zeneca (Cheshire, UK).

### Cell culture

MIN6 β cells were grown to 80–90% confluence in Dulbecco's Modified Eagle Medium (DMEM) containing 10% (v/v) FBS, 1% (v/v) penicillin/streptomycin. Cells were incubated for periods and with the appropriate effectors as indicated in the figure legends. Following appropriate incubation cells were washed twice with ice cold phosphate-buffered saline (PBS) prior to lysis in buffer containing 50 mM TRIS pH 7.4, 0.27 M sucrose, 1 mM Na-Orthovanodate pH 10, 1 mM EDTA 1 mM EGTA, 10 mM Na-β-glycerophosphate, 50 mM NaF, 5 mM Na-pyrophosphate, 1% (v/v) Triton X-100, 0.1% 2-mercaptoethanol and protease inhibitors. Whole cell lysates were centrifuged at 4000 *g*, 4°C for 10 min and stored at −20°C after being snap frozen in liquid nitrogen.

### Analysis of insulin release

MIN6 β cells were serum starved for 1 h in Krebs Ringer Hepes (KRH) buffer (10 mM Hepes, pH 7.4, 129 mM NaCl, 5 mM NaHCO3, 4.8 mM KCl, 1.2 mM KH_2_PO_4_, 1.2 mM MgSO_4_, 2.5 mM CaCl_2_ and 0.1% (w/v) BSA) prior to the addition of KRH buffer+glucose (3 or 16 mM) or an amino acid mix containing a nominal amount of glucose (3 mM glucose, 10 mM leucine and glutamine) for a further 1 h before analysis. After appropriate cell incubations and stimulation, 100 µl of the supernatant was removed and transferred into two fresh 96 well plates. The media was then diluted 1∶40 with KRH buffer (without BSA) and analysed for insulin content using an insulin ELISA kit. Unknown samples were calculated from a standard curve generated from known insulin standards supplied with the kit.

### Caspase-Glo 3/7 assay

Apoptosis was assessed using a Caspase-Glo 3/7 assay kit from Promega. MIN6 β cells were seeded into a white-walled 96-well plate and incubated with the appropriate effectors. Cells were washed and 100 µl of serum free DMEM media added to each well. The Caspase-Glo substrate and buffer were mixed together thoroughly and 100 µl was then added to each well and left for up to 3 h. The luminescence generated by the assay is proportional to the amount of caspase activity present, allowing quantification of apoptotic activity.

### Analysis of cellular ceramide and DAG content

After appropriate cell treatments, MIN6 β cells were extracted from plates using a rubber policeman in ice-cold PBS for analysis of ceramide and DAG using the DAG kinase method that relies on the kinase catalysing the formation of ceramide 1-phosphate and phosphatidic acid, respectively. Briefly, lipids were extracted from a cell aliquot containing 400 µg protein by the addition of chloroform/methanol (1∶2, v/v) with the phases being broken by the addition of chloroform and 1 M NaCl. The organic phase was separated, dried and used as substrate for the DAG kinase assay for measurement of ceramide and DAG content as described previously [52].

### SDS-PAGE and immunoblotting

Cell lysates (30–50 µg protein) were reconstituted in Laemmli buffer and subjected to SDS-PAGE on 10% resolving gels and transferred onto immobilon-P membranes as described previously [53]. Membranes were probed with primary antibodies of interest and subsequently detected using either horseradish peroxidase (HRP)-conjugated anti-rabbit IgG or anti-mouse-IgG (New England Biolabs). Membranes were visualized using enhanced chemiluminescence (Pierce, Northumberland, UK) on Konica Minolta x-ray medical film (Staffordshire, UK).

### 4′-6-Diamidino-2-phenylindole (DAPI) staining of cell nuclei

Cells were seeded onto 0.1 mm coverslips placed in a 6 well plate, and incubated with palmitate for times and at concentrations indicated in the figure legends. After the appropriate period of incubation with palmitate cells were washed three times with PBS. Cells were fixed by addition of 4% (w/v) paraformaldehyde (PFA) and left for 10 min. The PFA was removed and fixed cells washed three times with PBS before addition of 100 mM glycine for 15 min. The fixed cells were washed again with PBS three times and incubated with 50 µl of a diluted DAPI stock (10 µg/ml) for 15 min. The coverslips were then washed a further three times with PBS and mounted onto microscope slides. DAPI fluorescence was viewed and images captured using an inverted fluorescence microscope (Leica DM IRB, Leica, UK). Individual nuclei of adherent cells from randomly selected fields of view were counted from at least three separate experiments.

### Statistical analysis

One way analysis of variance (ANOVA) followed by a Newman-Keuls post test, was used to analyse statistical significance. Data analysis was performed using GraphPad prism software and considered statistically significant at values of *P*<0.05.

## Supporting Information

Figure S1 **SPT is expressed in MIN6β cells and its activity is sensitive to AZ422.** MIN6β cells were incubated in the absence and presence of 10 µM AZ422 for 48 h. Cells were then harvested and total membranes separated. (A) Membranes were immunoblotted for SPT-LCB1, SPT-LCB2 and the α1 subunit of Na/K ATPase for total protein. The blot confirms the expression of SPT within MIN6β cells. (B) In vitro SPT activity was analysed as described previously [28]. Briefly, 80 µg of total membranes were taken and the SPT reaction buffer added (100 mM HEPES pH 8.3, 2.5 mM EDTA pH 7.0, 5 mM DTT, 50 µM Pyridaoxal Phosphate, 1 mM serine, 1 µCi [^3^H]-serine/reaction and 10 µM AZ422 where indicated). Addition of palmitoyl-CoA initiated the assay (H_2_O substituted for palmitoyl-CoA in the control background sample). The reaction was allowed to proceed for 10 min at 37°C and terminated by the addition of 0.5 N NH_4_OH. Lipids were extracted using chloroform/methanol added to break the phases. NH_4_OH and sphinganine was added to act as a carrier for the lipid product generated. The resulting mixture was vortexed thoroughly and centrifuged. The upper aqueous phase was carefully removed and the lower organic phase, which contained the lipid products was washed twice with H_2_O. About 50–70% of the organic phase was vacuum-dried and the dried lipids reconstituted in 6.5∶1∶1 (v/v/v) chloroform/methanol/acetic acid and radioactivity assessed. Bars represent mean ± SEM from 3 separate experiments the asterisk signifies a significant difference between the indicated bars (P<0.05).
(PDF)

Figure S2 **Palmitate inhibits glucose-induced calcium oscillations.** MIN6 β cells were incubated with 0.4 mM palmitate for 48 h. Glucose was then added for 1 h for stimulation before assessment of calcium content. Briefly, during the 1 h glucose stimulation, cells were also incubated with 5 µM Oregon Green Bapta-1 probe (Molecular Probes, Invitrogen). After 1 h cells were washed 3× with KRH buffer and then viewed using a fluorescence microscope. Movies were taken over a 2–3 min time period but only the first 12 seconds are shown here to simplify the graph. Each time point is the average of 7 different cells but the error bars were removed, again for ease of view. Area under the curve analysis for each of the three conditions shown in the figure was performed using GraphPad prism and this analysis is presented in the table.
(PDF)
