# Supplementary figures and images for: Chronic Effects of Palmitate Overload on Nutrient-Induced Insulin Secretion and Autocrine Signalling in Pancreatic MIN6 Beta Cells
Source: PLoS One. 2011 Oct 5;6(10):e25975. doi: 10.1371/journal.pone.0025975 (PMC3187833; doi:10.1371/journal.pone.0025975)

**Fig. S1.**

**(A)**

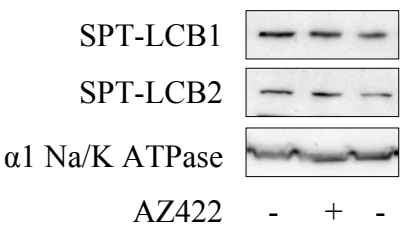

**(B)**

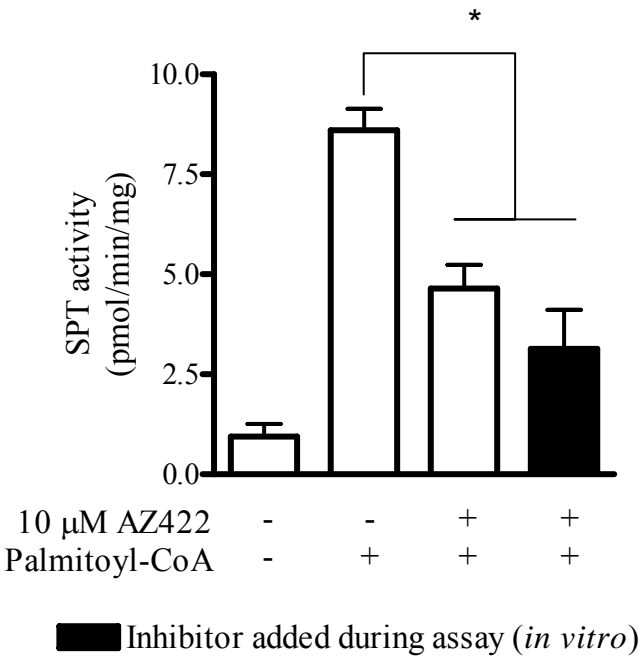

Supplement: Figure S1 — SPT is expressed in MIN6β cells and its activity is sensitive to AZ422. MIN6β cells were incubated in the absence and presence of 10 µM AZ422 for 48 h. Cells were then harvested and total membranes separated. (A) Membranes were immunoblotted for SPT-LCB1, SPT-LCB2 and the α1 subunit of Na/K ATPase for total protein. The blot confirms the expression of SPT within MIN6β cells. (B) In vitro SPT activity was analysed as described previously [28]. Briefly, 80 µg of total membranes were taken and the SPT reaction buffer added (100 mM HEPES pH 8.3, 2.5 mM EDTA pH 7.0, 5 mM DTT, 50 µM Pyridaoxal Phosphate, 1 mM serine, 1 µCi [3H]-serine/reaction and 10 µM AZ422 where indicated). Addition of palmitoyl-CoA initiated the assay (H2O substituted for palmitoyl-CoA in the control background sample). The reaction was allowed to proceed for 10 min at 37°C and terminated by the addition of 0.5 N NH4OH. Lipids were extracted using chloroform/methanol added to break the phases. NH4OH and sphinganine was added to act as a carrier for the lipid product generated. The resulting mixture was vortexed thoroughly and centrifuged. The upper aqueous phase was carefully removed and the lower organic phase, which contained the lipid products was washed twice with H2O. About 50–70% of the organic phase was vacuum-dried and the dried lipids reconstituted in 6.5∶1∶1 (v/v/v) chloroform/methanol/acetic acid and radioactivity assessed. Bars represent mean ± SEM from 3 separate experiments the asterisk signifies a significant difference between the indicated bars (P<0.05). (PDF) [file pone.0025975.s001.pdf]
