# Supplementary material for: Chronic Effects of Palmitate Overload on Nutrient-Induced Insulin Secretion and Autocrine Signalling in Pancreatic MIN6 Beta Cells
Source: PLoS One. 2011 Oct 5;6(10):e25975. doi: 10.1371/journal.pone.0025975 (PMC3187833; doi:10.1371/journal.pone.0025975)
Supplement: Figure S2 — Palmitate inhibits glucose-induced calcium oscillations. MIN6 β cells were incubated with 0.4 mM palmitate for 48 h. Glucose was then added for 1 h for stimulation before assessment of calcium content. Briefly, during the 1 h glucose stimulation, cells were also incubated with 5 µM Oregon Green Bapta-1 probe (Molecular Probes, Invitrogen). After 1 h cells were washed 3× with KRH buffer and then viewed using a fluorescence microscope. Movies were taken over a 2–3 min time period but only the first 12 seconds are shown here to simplify the graph. Each time point is the average of 7 different cells but the error bars were removed, again for ease of view. Area under the curve analysis for each of the three conditions shown in the figure was performed using GraphPad prism and this analysis is presented in the table. (PDF) [file pone.0025975.s002.pdf]

Fig. S2.

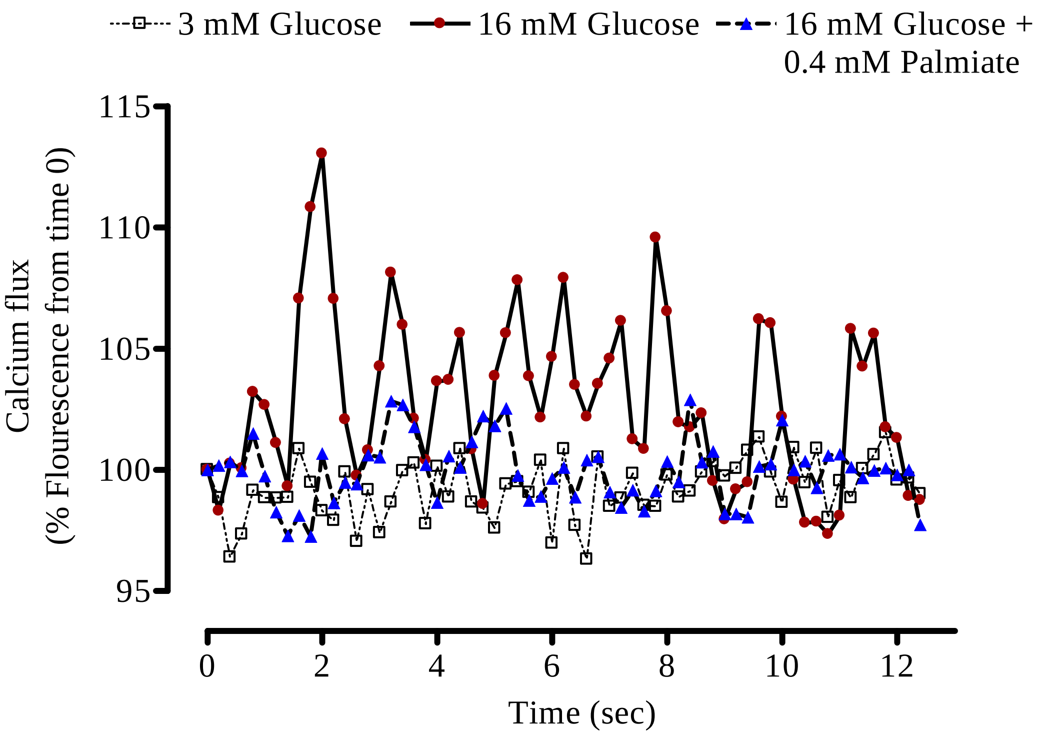

	3 mM Glucose	16 mM Glucose	16 mM Glucose + 0.4 mM Palmitate
Baseline	100%	100%	100.0
Area of positive peaks	1.317	38.55	5.300
Area of negative peaks	10.99	3.134	5.803
Net Area	-9.674	35.41	-0.5030
Total Peak Area	12.31	41.68	11.10
